# Exploring the Application Sphere of the Internet of Things in Industry 4.0: A Review, Bibliometric and Content Analysis

**DOI:** 10.3390/s22114276

**Published:** 2022-06-03

**Authors:** Raman Kumar, Sita Rani, Mohammed Al Awadh

**Affiliations:** 1 Department of Mechanical and Production Engineering, Guru Nanak Dev Engineering College, Ludhiana 141006, Punjab, India; sehgal91@yahoo.co.in or; 2 Department of Computer Science and Engineering, Gulzar Group of Institutions, Khanna 141401, Punjab, India; 3 Department of Industrial Engineering, College of Engineering, King Khalid University, P.O. Box 960, Abha 61421, Saudi Arabia; mohalawadh@kku.edu.sa

**Keywords:** Internet of Things, Industry 4.0, bibliometric analysis, content analysis, VOSviewer, Biblioshiny, review

## Abstract

This paper aims to comprehensively review 891 documents in the Scopus database about Internet of Things (IoT) in Ind 4.0 to understand the historical growth, current state, and potential expansion trend. From 2014 to 2020, a systematic methodology gathered information on IoT in Ind 4.0 documents in various Scopus databases. The relevant IoT research in Ind 4.0 documents is provided, and their types, publications, citations, and predictions are discussed. The VOSviewer 1.16.6 and Biblioshiny 2.0 applications display IoT status in Ind 4.0 publications for visualization research. The citation review aims to find the most prominent and influential authors, sources, papers, countries, and organizations. For citation analysis and ranking, document selection criteria were well defined. The author keywords, index keywords, and text data content analysis were used to identify the hotspots and development trends. The yearly IoT in Ind 4.0 article publications presented a speedily increasing trend, and a curve was fitted employing an exponential function. The paper “Intelligent manufacturing in the context of Industry 4.0: a review” was rated first with 754 citations. With 1629 citations, the “International Journal of Production Research” was ranked #1 along with Wan J. The South China University of Technology in Guangzhou, China, was placed first along with the United States as the most prolific and influential country. ‘Industry 4.0’ appeared the first time in 2014 with an application of IoT in Ind 4.0 with an overall appearance of 528, followed by the ‘internet of things’ in 2015, three times with a total count of 220 up to 2020. The IoT in Ind 4.0 assessment and bibliometric analysis intended to provide intellectuals a broad perspective working in IoT in Ind 4.0. Scholars should understand the hotspots in this area as soon as possible. This is also the first paper to thoroughly use bibliometric research to examine the IoT in Ind 4.0 literature. It will assist researchers of IoT in Ind 4.0 in expanding their knowledge and quickly comprehending the development status and pattern.

## 1. Introduction

The term “Internet of Things” (IoT) was introduced by Kevin Ashton in the year 1999 [1]. However, the concept of the IoT evolved over many years. Some different definitions were proposed by various researchers from time to time [2,3,4], but significant developments in this technology have only been observed in the previous few years [5]. It is acquiring massive admiration in the era of wireless communication. In the last decade, it has garnered much attention by showcasing its aim of connecting global infrastructure by catering all time and all place connectivity [6,7] to smart objects. Another aspect of the IoT facilitates thing–thing, human–human, and human–thing connectivity by allocating a unique identification code to each device on the network [8]. Key enabling technologies for IoTs are sensors, actuators, communication networks and protocols, and distributed stacks of intelligent objects.

**Figure 1:**
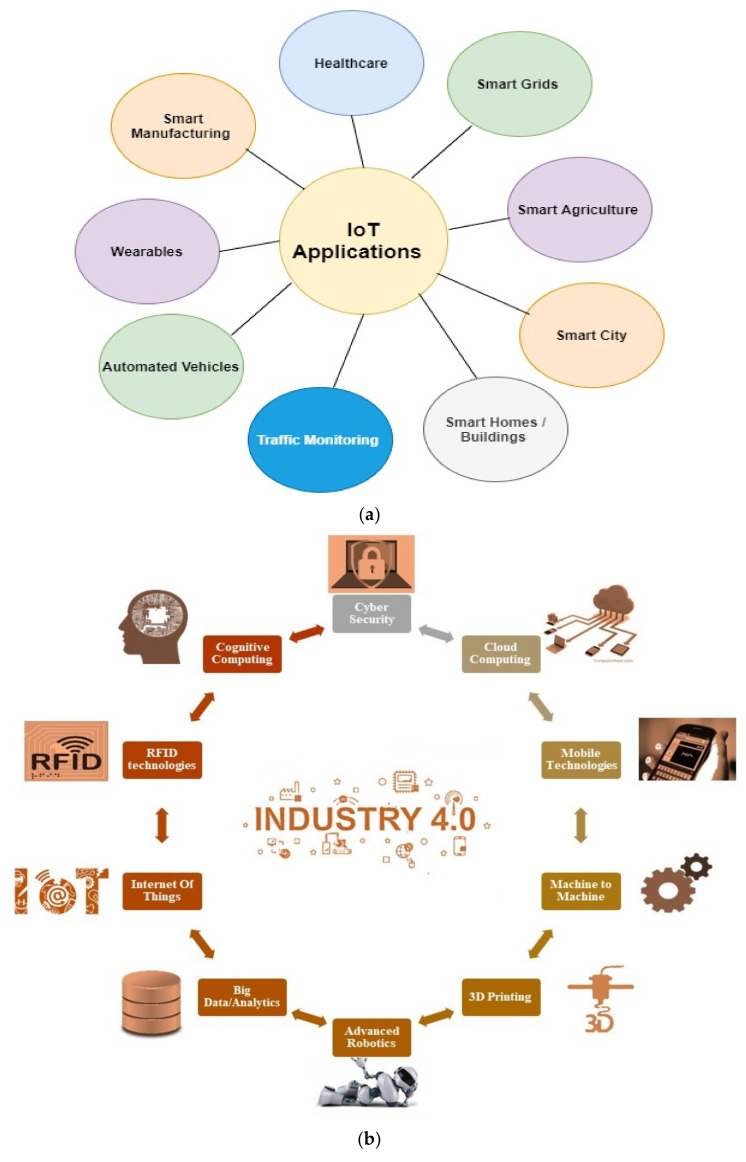
(**a**) Essential application area of IoT, (**b**) Industry 4.0: convergence of technologies and industrial processes.

Consequently, the Internet of Things is observed as networks of smart devices, also called “things”, connected in cyberspace. These smart devices collect, analyze, process, and transfer data to furnish various amenities to the users [8,9]. A considerable volume of data is assembled from multiple day-to-day activities [10]. Deployment of these operations is realized with the strong merger of the technology with other progressive knowhow such as Artificial Intelligence, Machine Learning, Big Data, Communication Networks (i.e., WSN), Cloud Computing, and so on [11,12]. The Internet of Things has captured different domains such as healthcare, smart cars, agriculture, smart homes, smart cities, smart industrial processes and operations, traffic management systems, smart grids and metering systems, and much more very quickly. By 2025, there will be approximately 50 billion smart devices worldwide comprising phones, cameras, smartwatches, TVs, Cars, smart AR/VR devices, healthcare devices, industrial equipment, etc. [13,14]. Some of the essential application areas of the Internet of Things are depicted in Figure 1a.

In recent years, IoT has drawn considerable interest from researchers. However, the heterogeneity of the smart devices interconnected in the framework of IoTs is a big challenge to analyze and process the data gathered in different formats. Smart things connected in cyberspace are also expected to be stacked with additional computational features to perform complex tasks [7]. Another major issue with the functioning of IoT is interoperability.

The new industrial revolution named “Industry 4.0” was first discussed in 2011 in Germany during the Hannover Trade Fair [15]. It is the fourth industrial revolution after Industry 1.0, 2.0, and 3.0 [16]. In the beginning, Industry 4.0 was launched to augment the probable of the manufacturing industry [17,18]. It comprises the integration of Information and Communication Technology (ICT) with the industrial framework to enhance the automation and digitization of the different industrial processes [19]. Frank et al. [20] discussed that technologies incorporated in the Industry 4.0 framework are deployed in two separate layers. Layer 1, also termed “front-end technologies” at the center of the framework, aims to transform the manufacturing process using recent technologies. The main features supported by this layer are smart supply-chain management, smart working, smart manufacturing, and smart products. Layer 2, comprising “base technologies” constitutes IoTs, cloud, big data, and analytics. This layer provides intelligence and connectivity to Layer 1 to provide various services. This layer differentiates the concept of Industry 4.0 from all previous industrial revolutions. Industry 4.0 was devised for a paradigm shift from dominant machine processes to digital manufacturing processes [19]. This new era of the global industrial revolution impacts the industrial structure, demand for goods, and competition rules. Competition rules are influenced as business models of digitized organizations are customized as per their needs [21]. Industry 4.0 is a novel manufacturing era in which integrated digital technologies permit organizations to provide advanced digital solutions to cater to customers’ needs. It also caters to the needs of business players in terms of reduced costs (workforce and material) and more productive production processes. Industry 4.0 is aimed at a vision of smart manufacturing with the integration of advanced technologies to deliver quality goods and services [22]. Industry 4.0 is realized using several technologies with industrial processes, shown below in Figure 1b.

Industry 4.0 comprises robotization, automation of manufacturing systems, and digitization of different industrial techniques, incorporating cyber–physical systems, vertical and horizontal integration of industrial subsystems, product and service customization, etc. [23]. Although initially Industry 4.0 was incepted to revolutionize the manufacturing processes [20], now it is benefiting several industrial sectors such as agriculture, food industry [15], steel industry [23], clinical research and healthcare [24], textile industry, pharmaceutical industry, paper industry, smart homes, and different types of the service industry.

There are several technological amalgamations for the proliferation of Industry 4.0. The internet of things is one of the key role players in this change [25]. In other words, the Industry 4.0 concept is realized mainly due to the deployment of IoT in various industrial processes [26,27]. Along with IoTs, the amalgamation of other advanced technologies such as cyber–physical systems (CPS), IoS, etc., facilitates the production of customized products [28]. Different types of equipment and devices are planted with radio-frequency identification (RFID) tags, sensors, actuators, and other intelligent devices in the industrial environment. These smart elements planted in the various IoT/smart devices play a crucial role in this industrial paradigm shift, aiding machine-to-machine communication and contributing toward a more automated industrial framework. IoTs facilitate more efficient data collection and transfer than cellular networks in an industrial environment [19]. This provides an improved understanding of various industrial processes and helps to make them more economical, efficient, and sustainable [29]. IoT plays a crucial role in many modern industrial applications, e.g., smart factories, digitized manufacturing, smart supply chain, logistics, etc. IoT can be integrated with Blockchain technology to ensure a higher level of security for industrial processes [30].

The domains of transformations in processes and operations with the integration of IoT devices in Industry 4.0 were reviewed. The authors presented the Industry 4.0 framework from the three aspects, i.e., automation, digitization of the processes, and connectivity of the various sites. Various constituents of Industry 4.0, i.e., smart factories, smart cities, cyber–physical systems, smart products, etc., are also discussed [31]. Okano (2017) discussed IoTs as the most speedily flourishing technology of the era of wireless communication and an ushering technology for Industry 4.0. Authors stated that IoTs were initially integrated into the manufacturing vertical and services in the modern industry [27]. Manavalan and Jayakrishna (2018) discussed the challenges faced by supply chain organizations and the complexity of the constituent processes. In this review, the authors present various opportunities to use IoTs to support a sustainable supply chain in the Industry 4.0 framework [28]. Xu et al., (2018) highlighted that Industry 4.0 originated from integrating various advanced technologies from different domains such as cloud computing, artificial intelligence, machine learning, cyber–physical systems, IoT, and additional business management processes. The authors provided an abstract role of all these technologies in the 4th industrial revolution. The authors also presented their survey’s research challenges and future directions [32]. Zhang et al., (2019) reviewed integrating different advanced technologies in Industry 4.0. This review discussed IoTs and Blockchain technologies concerning the automated industry. The authors were especially impressed by the role of IoT devices in the industrial transformation [33]. Industry 4.0 is a new industrial phase that provides different technological solutions to various challenges faced in the industrial framework. In this survey, the authors presented the post-implementation effects of three Industry 4.0 technologies, i.e., IoT, big data, and cloud computing, in various manufacturing companies [20]. Aheleroff et al., (2020) carried out a case study and discussed the crucial role played by IoTs in Industry 4.0. The authors discussed IoTs as the key contributors to delivering high-quality services and enhanced efficiency at a reduced cost. IoT plays a vital role in gathering data in different industrial processes in the industrial environment [34].

Muhuri et al., (2019) presented a thorough review of Industry 4.0. Different application domains of Industry 4.0 are also discussed. The authors also demonstrated the growth observed in the various verticals of Industry 4.0 from 2012 to 2017 [35]. Ahmi et al., (2019) carried out a detailed analysis of Industry 4.0 published literature. It was highlighted that most of the research related to this domain was carried out in Germany. It also presents Industry 4.0 challenges and future research directions [36]. Kipper et al. [37] carried out a bibliometric analysis of the published work from 2011 to 2018. IoT, Big Data, and Cyber–Physical Systems were observed as the most frequently occurring technological words during this work. The authors’ highlighted the integration of technologies, administration, and deployment as the major challenges faced and can be considered for future research work. An analysis of sustainable supply chain in Industry 4.0 was presented and concluded Industry 4.0 as an emerging field of research as approximately 4500 terms evolved to carry out future work [38]. Bigliardi et al., (2021) carried out a detailed study on logistics in Industry 4.0. The authors claimed it to be the first review of its type in Industry 4.0. In this study, the most frequently occurring terms were IoTs, CPS, and Logistics 4.0. The authors proposed Industry 5.0, AI, and 4D printing as the burning future research areas [39].

### 1.1. The Implication of the State-of-Art Review

The review indicated that most studies were done in several research areas to use IoT in the Industry 4.0 approach based on a comprehensive literature assessment. However, only a few review studies in the relevant field of research were conducted during this era as per the document type choice for information obtained from the Scopus database. However, in different applications, no attempt was made to test the usefulness of the IoT technology in Industry 4.0. In addition, in the Industry 4.0 method, the current literature has not conducted a systemic IoT literature review. Thus, the study required the IoT in Ind 4.0 approach to be thoroughly analyzed.

There are many bibliometric studies undertaken to highlight the importance of IoT in different fields, such as IoT and the water supply chain [40], IoT-based management in agriculture [41], IoT, cyber–physical system and smart city [42], megatrends in society [43], smart education [44], IoT and RFID [45], IoT, communication technologies in smart meters [46], IoT, digital transition and sustainability [47], actuators in a smart city [48], entropy weights [49], and technological forecasting [50]. However, the review of bibliometrics revealed that no such analysis is available covering the application of IoT in Industry 4.0. Therefore, sophisticated visualization tools are critical in IoT applications in the Industry 4.0 sector, especially when considering a systematic literature review and content analysis. Thus, the study extensively planned to examine the IoT applications in the industry 4.0 literature for six years, from 2014 to 2020.

### 1.2. Research Questions and Intended Contributions of the Existing Review

Keeping in mind the research gap derived from the above analysis, the study focuses on one of the essential research questions:

“Do we need any Bibliometric Analysis of the Internet of Things in Industry 4.0?”

The answer to this research question is yes; the extensive literature survey on IoT in Ind 4.0 has revealed an absence of a systematic analysis of IoT in Ind 4.0 frequently utilized by the academic world, industries, and research scholars. Suppose the proposed study systematically evaluated the entire literature in the form of bibliometric analysis. In that case, it will augment the overall methodical yield of IoT in Ind 4.0 and a considerate assessment of current bibliometric shreds of evidence and scrutiny of countless reasons for the diverse and multi-disciplinary practice of IoT in Ind 4.0. This will also help young researchers and users to draw valid conclusions on IoT in Ind 4.0. Therefore, the study has proceeded ahead with a positive intention to underwrite elucidated narrative reviews, a systematic review of IoT in Ind 4.0, and offer the much-needed future scope of IoT in Ind 4.0. Therefore, the study has arranged the following research objectives based on this research question.

### 1.3. Objectives of the Present Review

The study is based on the following research objectives based on an extensive literature review, research gap, and research questions under present investigation.

(1) To conduct an extensive bibliometric analysis of the existing literature on IoT in Ind 4.0.
(2) To propose a relevant research outlook related to the application of IoT in Ind 4.0 describing its future scope.

The study has organized a comprehensive bibliometric and statistical analysis of IoT in Ind 4.0 with the help of available research tools and software discussed in the upcoming subsection. The bibliometric analysis helps users gain an exhaustive picture of the study topic of their interest. The word was invented by Alan Pritchard [51], who argued that it might be used in any study seeking to describe the texting mechanism. This type of quantitative analysis will make different investigators aware of a given subject’s area, supporting them in appreciating the topic’s important history, the vast diversity of progress that has been made in this field, and future study opportunities.

## 2. Materials and Methods

### 2.1. Research Design

The systematic mapping study used a research plan that examines the complete IoT literature on Industry 4.0 by utilizing research techniques and software well described by [52,53]. The study employed a two-stage structured approach for bibliometric analysis and graphical evaluations [54] of the published scientific literature [55]. In addition, the inquiry led to the full examination of diverse publishing houses on the accessible IoT application in Industry 4.0. A determined effort was made to prevent the lack of any key IoT papers in Ind 4.0.

Table 1 shows the data accumulating steps accomplished in Scopus. The data collection was completed in October 2021 and limited the collection to 2020. As a result, the keywords search resulted in 3907 documents, limited to English results with 3810 and 3433 remaining while excluding 2021, and then the search was limited to articles only and excluded some subject area results of 891 studies. Further, all bibliometric analysis was performed on 891 articles.

### 2.2. Inclusive and Exclusive Criteria

IoT in Ind 4.0, inclusion–exclusion criteria were used to choose the necessary content. First, the survey considered only articles published in English, removing the remainder. Secondly, the publishing year was an essential factor for exclusion, and the period between 2014 and 2020 was the research period. Table 2 encapsulates the more progressive elements of inclusion and exclusion.

The number of items selected was calculated using the keywords indicated in Table 1 of the search strategy, following the inclusion–exclusion criterion. Finally, 891 papers were included in the last portion of the study. To fully comprehend their steady growth in the present state and future expansion trends in several other disciplines, the research aimed to examine 891 IoT database Scopus IoT articles from 2014 to 2020. Therefore, the scope of the investigation was restricted to articles between 2014 and 2020, and all other findings were eliminated. Consequently, the Scopus database recommended 891 final readable related materials in the article publications based on the keywords examined.

### 2.3. Research Procedure and Software Aid

The relevant data were analyzed using bibliometrics, visualization, content, and citation analysis. In addition, VOSviewer and Biblioshiny software packages were used in the research. VOSviewer 1.6.16 is a software application created by researchers at Leiden University in the Netherlands, i.e., Nees van Eck and Ludo Waltman. This program supports engrossed text mining, constructive features, and visualization display. It also offers an integrated representation of different bibliometric information, networks, and research [56]. Another quantitative research program employed in the current investigation was Biblioshiny Version 2.0. This program was developed by Massimo Aria and Corrado Cuccurullo from Naples University Federico II, Italy. This tool can carry out a thorough bibliometric analysis, drawing up the necessary data matrices, useful in a quotation, co-referencing, the comprehensive and wide-ranging linkage between variables, cooperation, and Coward’s research [57]. Many equations were utilized to determine normalized link strengths, association strength, fractionalization, and clustering and to accomplish different network plots in VOSviewer software; they are well presented in [58].

### 2.4. Statistical Methods of Data Summarization

Relevant research of IoT in Ind 4.0 is shown in graphs and diagrams concerning documentation, publications, citations, and predictions. From the perspective of the growth of IoT in Ind 4.0 publications, the tools VOSviewer and Biblioshiny were used to analyze the visualization effects. This research recognizes the most fruitful and dominant writers, sources, publications, nations, and organizations. For citation analysis and ranking, the screening process of documents was established. Prominent authors, sources, publications, nations, and organizations were allocated the most high-ranking positions according to the total citations, the collection of publications, the average total citation, and the overall strength of the links. Finally, in Ind 4.0, IoT features hotspots and progressive trends in author keywords, index keywords, and text data analysis.

## 3. Scrutiny of Articles, Sources, Authors, Institutions, and Countries: Internet of Things in Industry 4.0 (IoT in Ind 4.0)

### 3.1. Landscapes of IoT in Ind 4.0 Articles, Yearly Prophecy, and Citations

**Figure 2:**
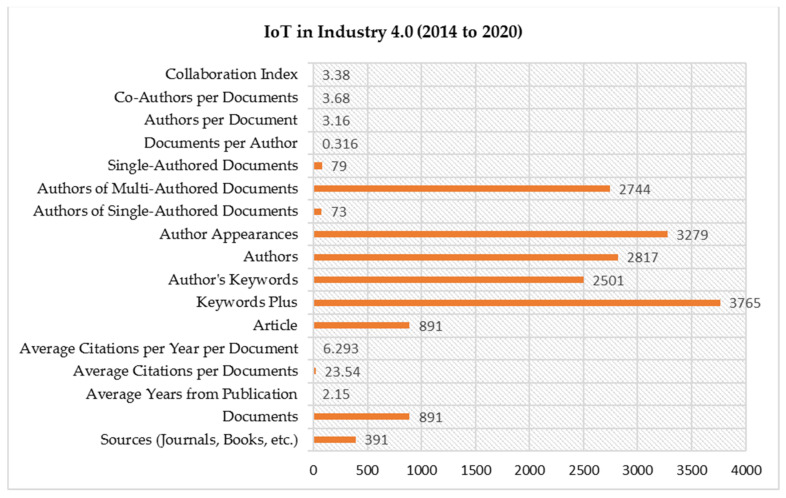
Vital Indication of IoT in Ind 4.0.

The prime evidence mined from the Scopus database on IoT in Ind 4.0 exploration data is shown in Figure 2. The IoT in Ind 4.0 records entailed 891 articles from 2014 to 2020 available in 391 journals. The composed articles had the following characteristics: 2.15 average years from a periodical, 23.54 average citations per article, and 6.293 average citations per year. All journals related to IoT in Ind 4.0 had 40,570 references. The mined statistics had only 391 sources. The 891 articles had 3765, 2501, 2817, and 3279 index and author keywords, authors, and appearances, respectively. Single authored articles totaled 79, with 0.316 and 3.16 authors per document. There were 3.68 co-authors per article with a 3.38 collaboration index.

**Figure 3:**
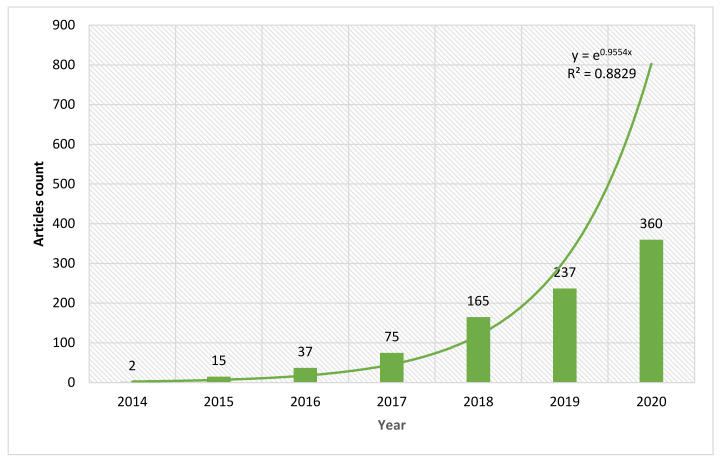
IoT in Ind 4.0 articles and prediction.

From 2014 to 2020, 891 articles were published on IoT in Ind 4.0, and the year-wise dispersal is shown in Figure 3. In total, 54 articles were published, with an average of 18 from 2014 to 2016 IoT in Ind 4.0 data collection. After that article periodical rate increased rapidly; from 2017 to 2019, 477 articles were published at an average of 159 articles. In 2020, 360 articles were published, and the publication trend increased. Since 2017, the article periodical frequency on IoT in Ind 4.0 amplified at a quicker stride. Overall, the annual article publications on IoT in Ind 4.0 displayed a rapidly intensifying drift since 2016. To show IoT in Ind 4.0 articles, a curve was fitted between time and reports published per annum, employing an exponential function, as displayed in Figure 3, showing the articles published annually; the R-square value was 88.25%.

**Figure 4:**
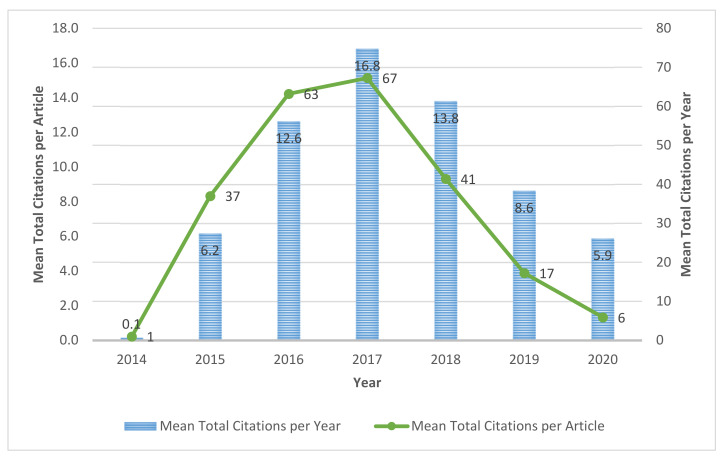
Distribution of mean citations of documents annually.

Only two articles were published in 2014 on IoT in Ind 4.0. Then, between 2014 and 2015, publishing speed in this area was virtually insignificant. The article mean citations per article and year are shown in Figure 4. After that, the publication in this area slowly increased by the year 2016. In 2016, 37 articles were published, and then this area received consideration attention by the researchers in IoT in Ind 4.0 and the corresponding regions. Finally, in 2019 and 2020, 237 and 360 articles were published, respectively, enriching the literature.

### 3.2. The Most Productive and Leading Article Citation Analysis

**Figure 5:**
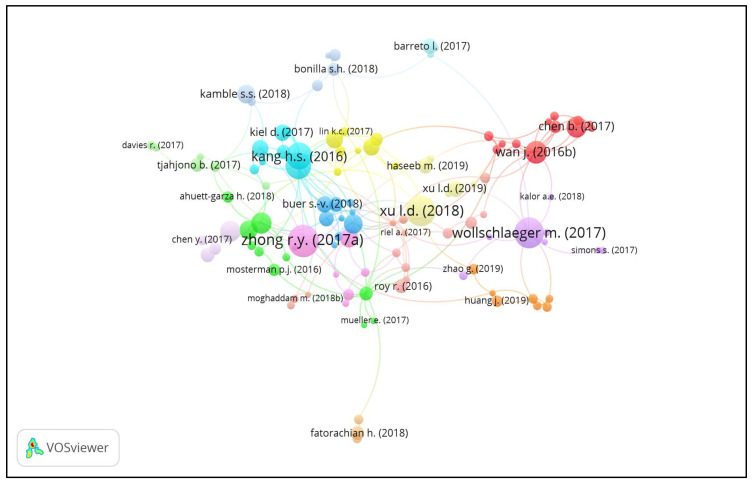
Article on IoT in the Ind 4.0 network.

The most cited articles on IoT in Ind 4.0 are explained and analyzed in this part [56]. The 891 articles on IoT in Ind 4.0 were analyzed using 30 citations, and 166 met the criteria. One hundred and twelve articles were connected and were divided into 16 clusters. Cluster 1 to cluster 16 consisted of 12, 10, 9, 8, 8, 8, 7, 7, 7, 7, 6, 6, 6, 5, 3, and 3 documents, respectively, as shown in Figure 5.

The consolidated citations were chosen as a ranking factor, with strong linkages and cumulative citations each year, and the ten leading articles are given in Table 3. The Zhong et al., (2017) [59] article titled “Intelligent manufacturing in the context of Industry 4.0: a review” ranked number one. The article has 754 citations, nine links, and 251.3 total citations per year. The article’s authors are affiliated with the University of Auckland, New Zealand and the University of Bath, United Kingdom. This article became productive and the leading article in IoT in Ind 4.0. and was published in the journal “Engineering”. The article titled “Industry 4.0: state of the art and future trends” ranked number one with ŤĈ/ŷ of 343 and second based on ŤĈ 686, authored by Xu et al., (2018) [32], and it was published in the journal “International Journal of Production Research”. The article titled “Industry 4.0 and the current status as well as prospects on logistics” ranked first based on ŤŁŜ of 15, fifth with 514 citations, and 171.3 ŤĈ/ŷ. This article was authored by Hofmann and Rusch (2017) [60] and it was published in the journal “Computers in Industry”. The prominent articles were cited more than 275 times, and the top five more than 500 times.

The findings of the citation study revealed that the most successful and excellent papers on IoT in Industry 4.0 processes were authored and analyzed properly. These findings indicate that the reader is ready to work on IoT for Industry 4.0. These academic publications summarize ideas from a wide range of papers. Often these papers cover a wider range. While most of these references or quotations indicate research that is critical, noteworthy, or pertinent to the critical evaluation by the usage of IoT in Ind 4.0, others describe literary works with a descriptive enough and wide background. The top 10 IoT documents in Ind 4.0 were published in leading scientific publications, as shown in Table 3. In addition, credit could not be awarded for their accessibility and the study findings given. Eminent scientists from the most recognized educational institutions around the globe have also submitted papers. Subsequently, they have garnered great respect and appreciation in their field of specialization. The top 10 quoted journals are from New Zealand, the UK, South Korea, Germany, Switzerland, Sweden, China, Brazil, and France. They all come from Germany and France. At the same time, they showed a strong potential for prompt marketing of inventions.

#### Literature Survey of Top 25 Cited Documents and Contributions to IoT in Ind 4.0

Zhong et al., (2017) presented a review on developments caused by integrating different technologies, i.e., Artificial Intelligence (AI), IoT, and Cloud Computing, in intelligent manufacturing techniques in Industry 4.0. Major achievements in Industry 4.0 are increased flexibility, greater scope of customization, enhanced productivity, time economical, and better quality of the products. Different technologies such as Information and Communication Technology (ICT), IoT, Big Data, Cyber-Physical Systems (CPS), and Cloud Computing were reviewed in this work. Furthermore, the authors presented the future development prospects in intelligent manufacturing by highlighting its contribution to the value addition of the products and services to substitute traditional manufacturing [59]. Xu et al., (2018) presented the states of industrial evolutions, i.e., from Industry 1.0 to Industry 4.0, and enabled technologies integrated into various industrial operations. The detailed presentation of the entire industrial revolution highly contributed to the popularity of this article.

The role of IoT, Cloud Computing, and CPS in Industry 4.0 was discussed. The authors highlighted the lack of availability of suitable technologies, protocols, and mechanisms to leverage the maximum benefit of Industry 4.0. An overview of security and privacy issues was also discussed. The study was concluded with various research challenges and possible future directions in the Industry 4.0 framework [32]. Wollschlaeger et al., (2017) impressed upon the role played by IoT and CPS in the fourth industrial revolution. IoT-enabled communication technologies in Industry 4.0 were discussed in this work. In addition, the authors discussed the role of 5G technology in industrial process automation in detail. The authors also presented a pathway to the 5^th^ Industrial Revolution [61].

Kang et al., (2016) presented a detailed review on smart manufacturing in Industry 4.0. Emerging research trends in smart manufacturing in countries such as Germany, the United States, and Korea are the key features of this work. The detailed discussion of the industrial revolution in developed countries gave the authors and researchers a new direction. The authors presented the basic concept and different technologies involved in smart manufacturing. The authors also discussed the expected future developments in smart manufacturing in Industry 4.0 [62]. Hoffman and Rusch (2017) highlighted the transformation that occurred in manufacturing the goods during the industrial revolution. In this paper, the domain of logistics management was reviewed. IoT, CPS, Internet of Services (IoS), and Smart Factories are presented as the main components of Industry 4.0. The authors also discussed the key implications and future prospectus of Industry 4.0 in logistics and talked about the uncertainty of the domain even after technological development [60]. Wan et al., (2019) discussed CPS and IoT as the key enabling technologies for Industry 4.0. The development caused by the Industrial Internet of Things (IIoT) was also highlighted. The authors also discussed integrating other advanced technologies such as cloud computing, wireless networks, big data analytics, and other smart devices with IIoT to deliver reliable and customizable services [67].

Frank et al., (2019) presented the basic concept and framework of Industry 4.0. The need to integrate various advanced technologies to cater to various digital solutions in an automated industrial environment was highlighted. IoT, Cloud Computing, and Big Data Analytics are the base technologies and smart manufacturing and smart supply chains as key industrial automation domains. The role of IoT in conjunction with other advanced technologies in these domains is predicted to play a vital role in future industrial development [20]. Chen et al., (2017) discussed the layered architecture of a smart factory. IoT, Big Data, and Cloud Computing are the key technologies in the smart industry framework. The need for intelligent devices, strong convergence of communication networks, and information-based manufacturing were highlighted as the major challenges the smart factory faces in Industry 4.0 [64]. Tao and Zhang (2017) discussed the role of ICT in the emergence of Industry 4.0. The authors highlighted the need to integrate physical and cyber work to realize the concept of smart manufacturing. A digital twin shop-floor was discussed in detail by exploring its key components, technologies, and major challenges faced in the domain. Finally, the authors discussed the different concepts in different countries for smart manufacturing using IoT, AI, and Big Data. The authors also talked about the limitations and challenges faced due to technology integration [65]. Moeuf et al., (2017) conducted a review on the administration techniques of SMEs in the Industry 4.0 environment. Different issues faced in production planning and management of SMEs in Industry 4.0 were presented in detail. Flexibility, reduced cost and time, better productivity, and quality were the main performance objectives in the new industrial era, achieved by integrating IoT, Big Data Analytics, Autonomous Robots, CPS, M2M Communication, and Cyber security [66]. Sanders et al., (2016) discussed Lean Manufacturing methodology with the competence to optimize various operations of manufacturing organizations. The authors highlighted the possibility of integrating Lean Manufacturing in Industry 4.0 to make the factories lean and smart. The authors also presented the challenges faced in this application area in the 4th industrial revolution [68]. Kamble et al., (2018) discussed IoT as one of the key contributors in Industry 4.0 to digitize various industrial processes. In this review, the authors’ main focus was the different technologies used and the sustainability of Industry 4.0 operations. IoT, CPS, Cloud Computing, Simulation and Prototyping, 3-D printing, and Cyber Security in industrial automation were discussed. From a sustainability aspect, the key areas discussed were economic stability, sustainable industrial processes, and the environment [69].

Boyes et al., (2018) presented IoT as the key technology to enable connectivity in industrial automation. The development of Industrial IoT (IIoT) and its relationship to CPS was discussed in detail. The authors also presented the research gaps and future directions to overcome those gaps. The significance of the security of industrial data while communicating in cyberspace was impressed upon in this article [70]. Muller and Voigt (2018) discussed the impact of the Internet of Things and Services (IoT) on the automation of various processes and mechanisms in the Industry 4.0 framework. It was also discussed that IIoT realizes complete connectivity in Industry 4.0.

Technical and economic aspects, opportunities, and challenges were reviewed for Industry 4.0 applications from a sustainability aspect [71]. Jabbour et al., (2018) conducted a review on Circular Economy (CE) in industrial applications. Different advanced technologies playing a key role in Industry 4.0 were also discussed. The fundamental aim of this review work was to explore the methods to increase the CE application stake in Industry 4.0 by integrating these two technologies [72]. Pereira and Romero (2017) reviewed basic concepts, technologies, and developments in Industry 4.0. In this paper, IoT, CPS, and their integration to improve industrial processes, productivity, market share and economy, develop new business strategies, and customize the work environment on Industry 4.0 were discussed [73]. Buer et al., (2018) discussed the increased popularity of Industry 4.0 concepts in academia and industry. This review was carried out to identify the relationship between Lean Manufacturing and Industry 4.0. The authors highlight that at the beginning of the development of the concept of Lean Manufacturing, many challenges were administered using Information and Communication Technology (ICT). It was also discussed that ICT in Lean Manufacturing caused new opportunities and potential developments in Industry 4.0. IoT and CPS were presented as the key technologies to realize distributed, reliable, automated, and flexible industrial systems [74].

Xu and Duan (2018) discussed Big Data and CPS as the key role players for resource optimization in the Industry 4.0 framework. Optimal resource utilization is one of the key factors in sustainable industrial development. The authors also addressed a lesser number of surveys where the integration of these two technologies in industrial automation has been discussed. In this work, the basic concept of big data, its characteristics, and the big data challenges of CPS are discussed in detail. Finally, the paper was concluded with future research challenges in the security domain of CPS in Industry 4.0 [75]. Kiel et al., (2017) discussed IoT and IIoT’s role in delivering reliable, economic, and sustainable Industry 4.0 applications. CPS was discussed as the key component of IIOT. The authors also presented the benefits and major challenges of integrating IIoT in various industrial processes. The authors compared three industrial sectors, i.e., electrical firms, machine industry, and automation, regarding benefits and challenges faced in IIoT applications [76].

Barreto et al., (2017) discussed the role of IIoT in the supply chain and presented it as the key reason behind logistics in Industry 4.0. In addition, the authors discussed various requirements and fundamental issues different organizations face in deploying fully functional Logistics 4.0. Possible future research directions in the deployment of technology equipped logistics were also presented in this paper [77]. Finally, Haseeb et al., (2019) studied the role of Industry 4.0 in increasing business sustainability. This survey was conducted for SMEs in Thailand. In this survey, IoT, CPS, Big Data, and Smart Factories emerged as the key technologies to promote sustainable business for SMEs. However, the survey was conducted only for SMEs, so observations cannot be generalized to other industries [78]. Aazam et al., (2018) impressed upon the industrial revolution caused by technological development. The authors mentioned IoT as the key technology for Industry 4.0 and also the base of IIoT. Using these two technologies loaded with different sensing devices and equipment, a huge volume of data is generated from diverse industrial processes. These data need to be processed locally to avoid delays and security issues, which is the fundamental reason to deploy Fog in IIoT. In this paper, the authors discussed the basic concepts of Fog Computing, IIoT, and Industry 4.0. Various advantages of integrating Fog Computing in industrial applications were also discussed. The authors concluded their work with the challenges of integrating Fog Computing in Industry 4.0 applications [79]. Yin et al., (2017) presented a review on the revolution of the production system from Industry 2.0 to Industry 4.0. Customer demand was characterized as the key player in the evolution of the production system over time. The authors also discussed the future possibilities of IoT-facilitated smart factories [80]. Schroeder et al., (2016) proposed a new model using Automation ML for Digital Twin. This model was proposed concerning using CPS for industrial manufacturing systems and delivering better quality product services in Industry 4.0 [81]. Sommer (2015) discussed Industry 4.0 as the evolution caused by smart devices and equipment. In the Industry 4.0 framework, SMEs face several challenges in terms of their competence, readiness, and potential to integrate the advanced technologies in various subsystems and processes. Due to these challenges, the German industry cannot take all benefits of industrial automation in the SME sector. However, this industry is planning and adopting several action plans to overcome these issues [82]. The technologies for possible integration, domains, and evolution of technologies are shown in Table 4 for top-cited documents related to IoT applications in Industry 4.0.

From the literature analysis of top-cited articles, the integration of IoT in different processes revolutionized the various domains in the Ind 4.0 environment focusing on different aspects such as the design, production processes, manufacturing, economy, services, sustainability, business strategies, supply chain, security, and communication of data, etc. in different types of organizations, viz. SMEs, the electrical and machine industry, etc. During the study, it was observed that major development is caused by the integration of IoT with other advanced technologies such as AI, big data, cloud computing, fog computing, blockchain, and CPS. CPS is observed as one of the key role players in this IoT era of industrial development. IIoT is an evolution of IoT by emphasizing smart industrial devices and equipment, conveniently making secure M2M communication possible. The confluence of IoT with other technologies offers economical, sustainable, and better-quality services to the users in the smart industry. It has also reasoned the optimal utilization of the industrial resources and secured transfer and management of industrial data gathered through different processes.

### 3.3. The Most Productive and Leading Journal Citation Analysis

**Figure 6:**
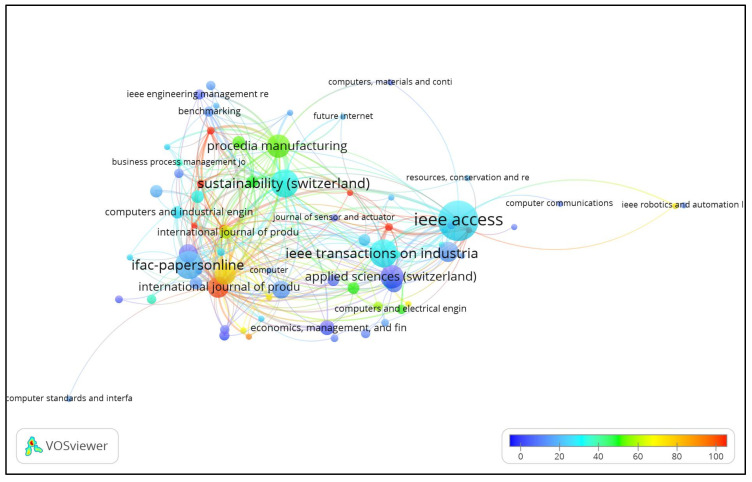
Journal overlay visualization based on 20 citations and two articles.

Journal overlay visualization based on 20 citations and two articles is shown in Figure 6. Out of 391 journals, 81 met the threshold. Seventy-three journals were connected and divided into thirteen clusters consisting of 9, 8, 8, 5, 5, 5, 4, 4, 4, 3, 3, 3, and 2 journals.

The top ten sources constructed based on the ŤĈ, NoA, ŤŁŜ, and average ÄĈ are shown in Table 5. The “International Journal of Production Research” ranked first when the ŤĈ was selected as a measure for the journal’s influence, with 1629 ŤĈ, and the first paper on IoT in Ind 4.0 was published in 2018. The IEEE Access ranked first based on NoA (53), second based on 1470 ŤĈ, and nine based on ÄĈ. The Computers in Industry ranked second with a 71 ŤŁŜ and third with 1247 ŤĈ. The PSY, ĬF, and CS of the top ten journals are also shown in Table 5. Out of the top 10 sources, “IEEE Industrial Electronics and Magazine” has an ĬF of 13.593 with 710 ŤĈ in IoT in Ind 4.0.

**Figure 7:**
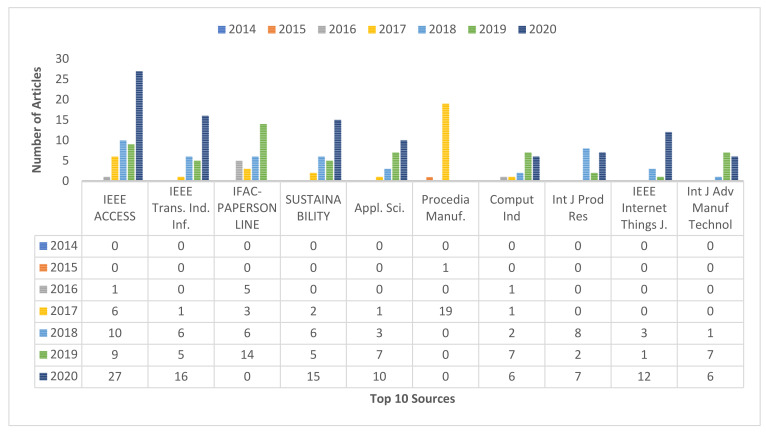
Top ten source dynamics.

Figure 7 depicts the top ten journal-published articles on IoT in Ind. 4.0 from 2014, the starting year up to 2020. The journals published IoT-based research in industry 4.0 in 2016, which topped the NoA (53). The journals published a maximum of 27 articles in 2020 with an average of eight research publications. IEEE Transactions on Industrial Informatics, IFAC-PapersOnLine, and Sustainability (Switzerland) published a maximum of 28 articles with an average of four articles, and they published the highest NoA of 16 in 2020, 14 in 2019, and 15 in 2020, respectively. They started publishing in 2017, 2016 and 2017, respectively.

### 3.4. The Most Prolific and Dominant Author Citation Analysis

**Figure 8:**
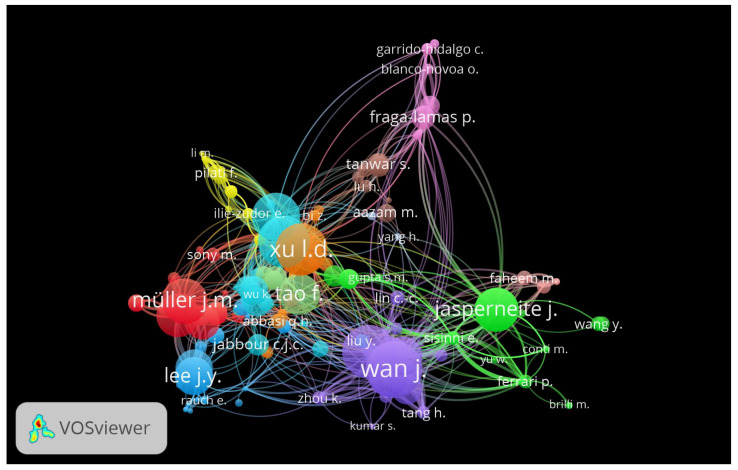
Author network of IoT in industry 4.0.

**Figure 9:**
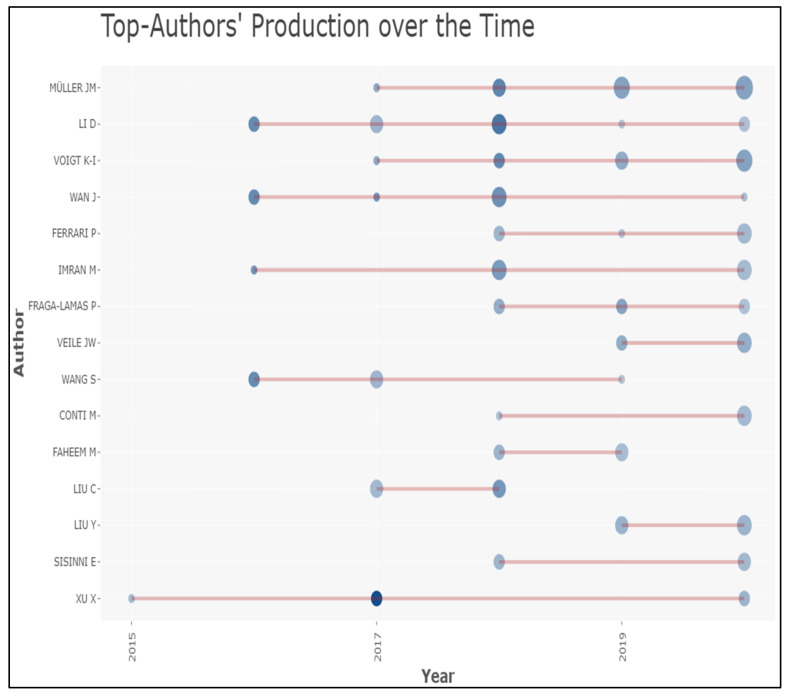
Topmost author citations and publication status by year.

The most prolific authors were selected, having a minimum of two articles and 20 citations. Figure 8 shows the 172 authors’ network of IoT in industry 4.0, out of 2817, divided into twelve clusters. The diverse rankings of the authors are shown in Table 6. Wan J. ranked first, based on 1031 ŤĈ, second based on ŤŁŜ of 132, and third with 8 articles. Wan J. is affiliated with the School of Mechanical and Automotive Engineering, South China University of Technology, Guangzhou, with an h-index of 7, g-index of 8, and m-index of 1.167. Wan J. started publishing on IoT in Ind 4.0 in 2016, and the year-wise NoA and ŤĈ evolution can be understood in Figure 9. NoA has a dark blue bubble, and a light blue bow is ŤĈ per year. The second most prolific and dominant author was Li D., based on ŤĈ.

#### Lotka’s Function: Author Script Explanation

**Figure 10:**
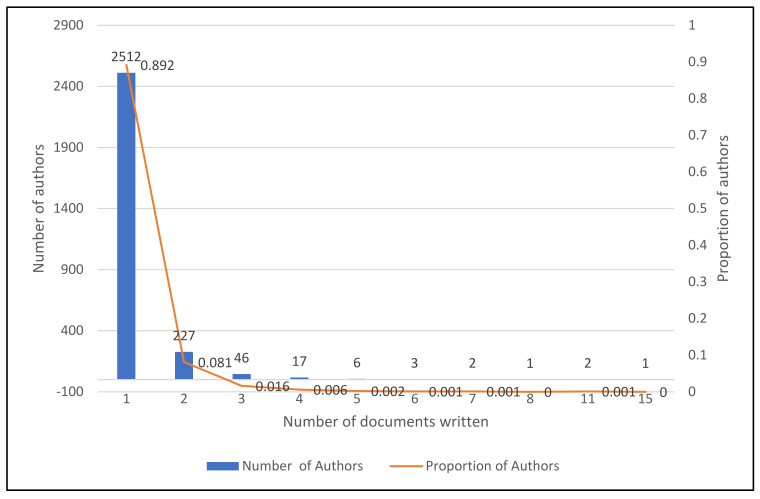
Lotka function representation.

The law of Lotka is a series of Zipf’s special laws named for Alfred J. Lotka. It specifies how often writers publish in a certain field. It emphasizes that a portion of the total number of academics who participate in one year is the number of scholars contributing—consequently, the frequency of production declines with the number of publications [83,84]. Figure 10 shows the number of writers who collaborated on a particular article. Two thousand, five hundred and twelve authors wrote one article with a proportion of authors of 0.8992, and two papers were written by 227 authors with a proportion of 0.081. Forty-six authors wrote three articles with a 0.016 ratio of authors. Four documents were written by 17 authors, five by six, six by three, and seven by two authors, referred to in Figure 10. A solo author wrote the top ten 15 documents with zero proportion of other writers.

### 3.5. Citation Analysis of Organizations and Nations

“IoT in Ind 4.0” comprises 1888 organizations, and ranks were appointed for each organization, considering a minimum number of papers (2) and 20 citations. Out of the 45 organizations, rank was allocated based on ŤĈ, NoA, ŤŁŜ, and ÄĈ, as shown in Table 7. For example, South China University of Technology, Guangzhou, China, ranked first based on ŤĈ of 788, NoA equal to four, and ŤŁŜ of 17. Beihang University, Beijing, China, was second based on ŤĈ of 439 and first with ÄĈ of 220. The most prolific and dominant country was the United States, with 4820 total citations, 254 articles, and 178 total link strengths and ranked fourth based on average article citations of 19. Singapore was in the first position based on an average article citation value of 82.

**Figure 11:**
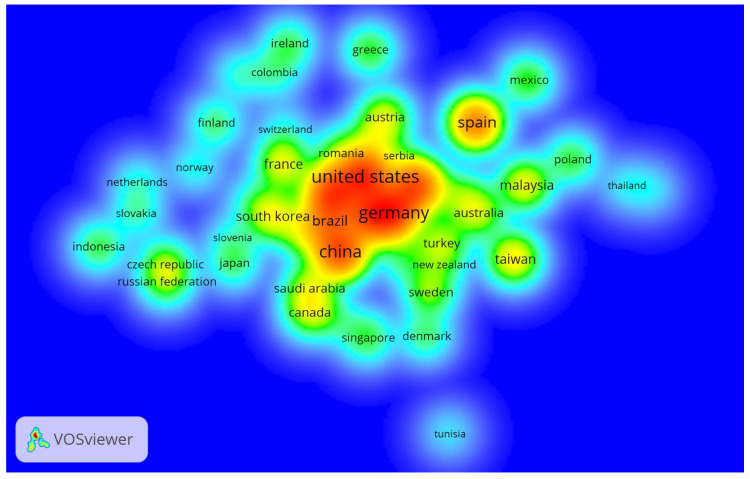
Nations: density visualization.

There are 101 nations engaged in IoT in Ind 4.0 research, with ranking considering a minimum of five documents for each nation and twenty citations. Out of 101 nations, 48 met the criteria and were divided into nine clusters consisting of 9, 9, 7, 7, 7, 4, 4, 3, and 3 nations. The density visualization of seven clusters is shown in Figure 11.

## 4. Co-Occurrence of Keyword and Content Analysis

The co-occurrence paradox discusses how some things exist simultaneously, such as keywords or index terms. The literature includes more information than the research, including organizations, publications, authors, and keywords. A quantifiable analysis of the co-occurrence structure exposes the implication of information and the knowledge that some items hide. Keywords can also be used to determine the parameters of the survey and control hotspots and phenotypic expression for the field of practice and study [85]. Content analysis is a method of study used in evaluating and coding textual content to provide reproducible and accurate information. Qualitative information may be turned into quantitative information such as papers, oral statements, and graphics by methodically examining words [56].

### 4.1. Co-Occurrence Analysis of Author Keywords in IoT in Ind 4.0

**Figure 12:**
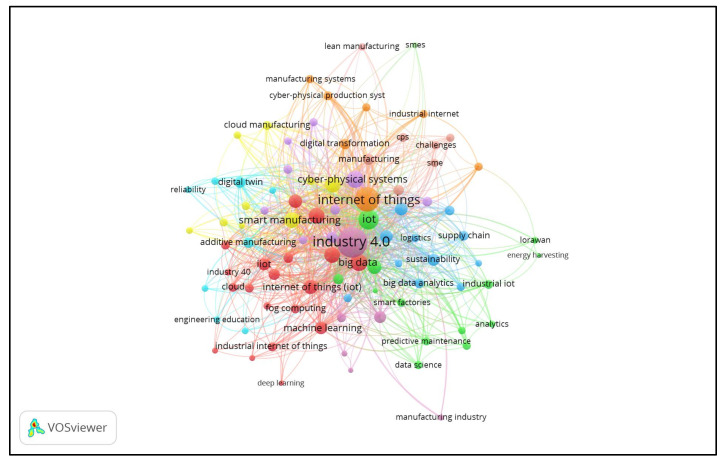
Author–keyword network visualization.

**Figure 13:**
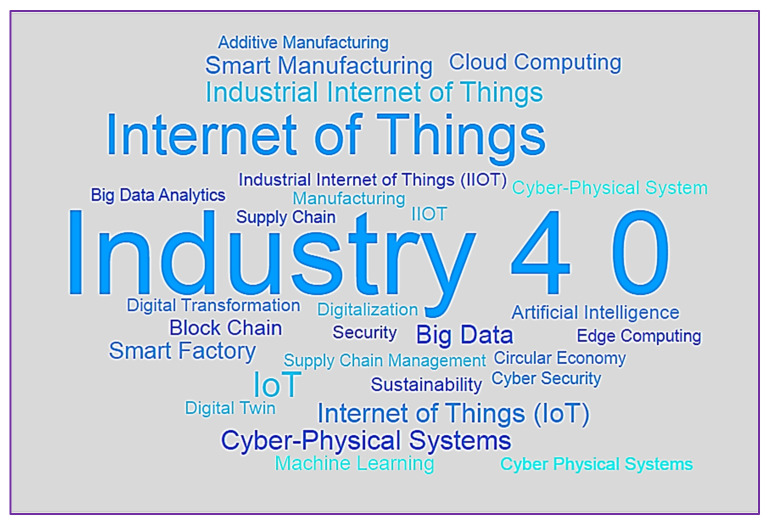
Word cloud of top-30 author keywords.

This section analyses the authors’ co-occurrence in IoT in Ind 4.0 from 2014 to 2020 based on Scopus datasets. Five co-occurrences were used for the IoT in Ind 4.0 to choose the keywords. There appeared to be 2501 author keywords and 90 relative to the five co-occurrence criteria of author keywords. The largest group of linked items comprised 90 keywords split into 11 clusters. The visualization of co-occurrences by the author–keyword network is displayed in Figure 12. The word cloud of the top 30 author keywords is shown in Figure 13 (Figure generated from: https://worditout.com/word-cloud/create, accessed on 25 May 2022).

Table 8 depicts the evolution of author keywords from 2014 to 2020. The term “Industry 4.0” first emerged in 2014, and since then, its use has grown exponentially. It had reached 219 by the end of 2020 and was featured 528 times in total, averaging 132 per year from 2014 to 2020. The author keyword internet of things first occurred in 2015, and its popularity grew steadily from there, peaking at 220 in 2020 with an average of 55. More than 15 times, the terms IoT, Industrial Internet of Things, and Cyber–Physical Systems appeared. Except for Industry 4.0, no one term from the top ten-word dynamics was utilized in 2014.

### 4.2. Index Keywords of IoT in Ind 4.0: Co-Occurrence Analysis

These are keywords selected by content providers and standardized according to the vocabulary available to the public. Contrasting the author’s keywords, synonyms, spelling, and plural forms are considered in the indexed keywords. An index term, a term, a topic or a descriptor is a term that encapsulates the core of a document’s subject matter in the collection of information. Index words provide a regulated language for use in bibliographic documents. The bibliographic controls are an inherent component of the libraries’ collection, management, and distribution.

**Figure 14:**
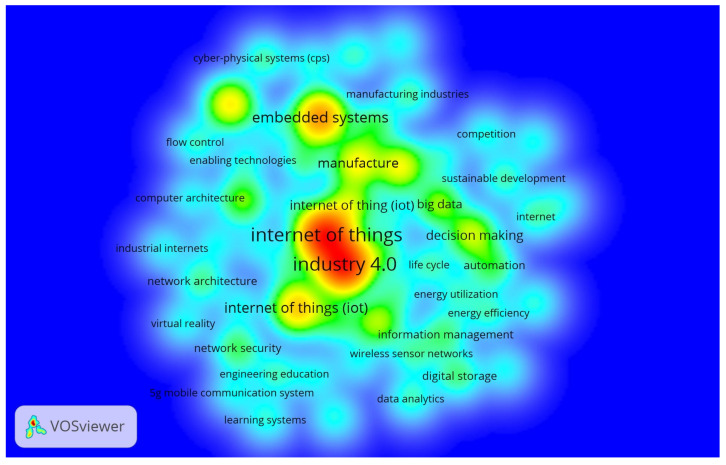
IoT in Ind 4.0 (15 index keyword co-occurrences).

**Figure 15:**
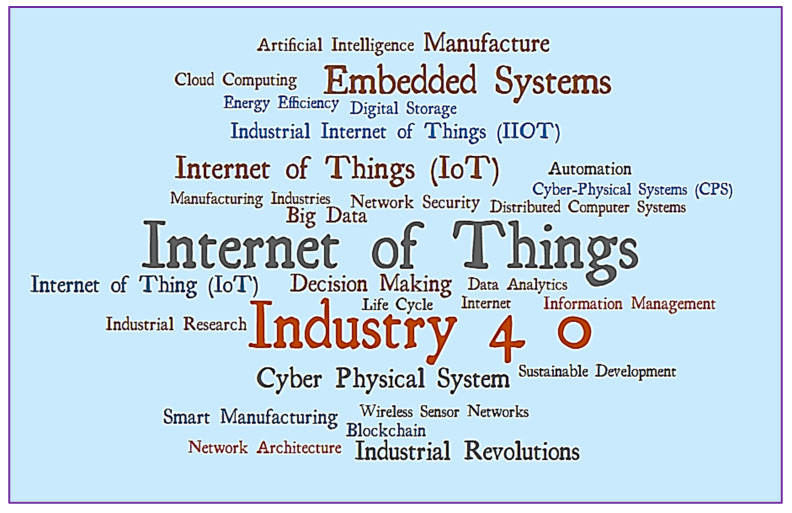
Word cloud of keyword-plus.

This portion describes the co-occurrence of index terms in IoT in Ind 4.0 between 2014 and 2020 for Scopus records. The index keywords for IoT in Ind 4.0 were determined based on a total of 15 cases. As a result, there were 3765 index keywords for IoT in Ind 4.0, of which 58 fulfilled the threshold for 15 co-occurrences. Thus, 58 index keywords were divided into five groups in the most thorough list of associated items. Figure 14 shows the index keyword density representation. Word dynamics of index keywords from 2014 to 2020 are illustrated in Table 9. The word cloud of the top 30 index keywords is shown in Figure 15 (Figure generated from: https://worditout.com/word-cloud/create, accessed on 25 May 2022).

### 4.3. Text Data Analysis of IoT in Ind 4.0

The VOSviewer software executes an exclusion method with a text data analysis and calculates each term’s relevance score. Words with higher relevance mean explicit topics addressed by text information throughout the text data extraction procedure. The phrases for the extraction of text data, on the other hand, tend to be less relevant and do not reflect a specific topic. Thus, phrases collected with insufficient relevance data are selected, and explicit and informative pieces are the key topics. About 40% of data collected are removed with poor relevance ratings [56]. The words in the title and abstract of the article are retrieved in textual analysis. Even with the binary technique and the relevance score, the title and abstract data retrieved from the article are analyzed [86].

**Figure 16:**
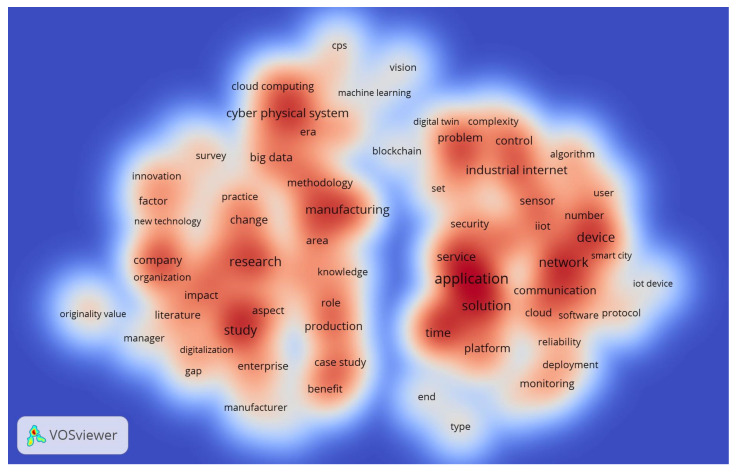
IoT in Ind 4.0: Visualization of 104 text terms.

Terms were selected based on 30 occurrences in the IoT text-data analysis in Ind 4.0. In Ind 4.0, 18,225 text-data words and 154 correspond to 30 events on IoT. Therefore, 60 percent of the relevant words were taken into account, with 92 based on the relevancy score. Figure 16 shows the display of the density of linked words. The largest range comprises 92 words grouped into clusters 1, 2, and 3, consisting of 52, 39, and one IoT in Ind 4.0 terms.

**Figure 17:**
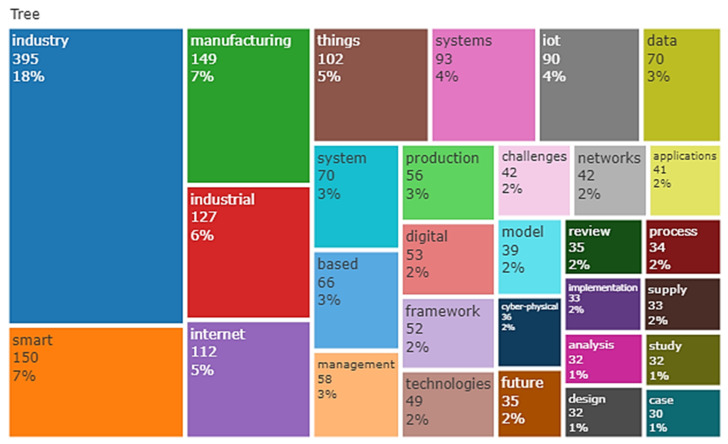
Tree plot of the top 30 title words.

**Figure 18:**
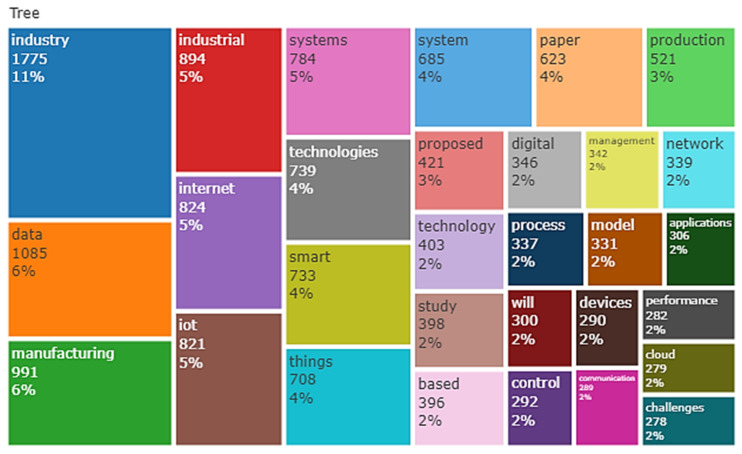
Tree plot of the top 30 abstract words.

Table 10 depicts IoT in Ind 4.0 text data, i.e., data extracted from the abstract and title of collected relevant documents. For example, the word “application” appeared 282 times with a relevance score (RS) of 0.407 from the top 25 terms. On the other hand, the term “design methodology approach” topped the list based on an RS of 8.432 with an occurrence (Occ) of 34. Furthermore, the terms “study”, “network”, “solution”, and “research” were in the top five based on the occurrence of 215, 210, 19, and 195, having relevance scores of 0.856, 1.230, 0.485, and 0.686, respectively. Finally, the terms “originality value”, “IoT device”, “manager”, and “protocol” were in the top five based on a relevance scores of 7.665, 3.906, 2.262, and 2.225, occurring 36, 41, 46, and 60 times, respectively. Thus, almost all of the top 25 terms used in the IoT in the Ind 4.0 database appeared around 100 times. Word dynamics of titles and abstracts of IoT in the Ind 4.0 database are depicted in Table 11 and Table 12, respectively. A tree plot of the top 30 title words is shown in Figure 17; ‘industry’ appeared 395 times with 18%, followed by ‘smart’ and ‘manufacturing’, i.e., 7%. The tree plot of the top 30 abstract words is shown in Figure 18; ‘industry’ appeared 1775 times, i.e., 11%, followed by ‘data’ and ‘manufacturing’, i.e., 7%.

### 4.4. Three Field Plots on IoT in Ind 4.0: Sankey Illustrations

A Sankey illustration is used to represent a flow from one subset to another. The linkages are called nodes, and the interconnections are called links. The ideal way to display Sankeys is to map different systems. Sankey diagrams are usually used in numerous networks and techniques as representations of energy or material flows. They represent quantitative details of flows, relationships, and transition. Sankey diagrams provide guided and weighted designs with weight characteristics that maintain the flow. At each node, the weights of the inflow are the same as the outcome [87].

Biblioshiny’s three-field plot visually evaluates the link between sources, nations, affiliations, index keywords, authors, journals, and author keywords, etc. Rectangular diagrams depict relevant elements with different descriptions using colors. The rectangle’s height links several factors such as nations, sources, renowned authors, writers’ and keywords. The larger the rectangle, the more interconnections between different components.

**Figure 19:**
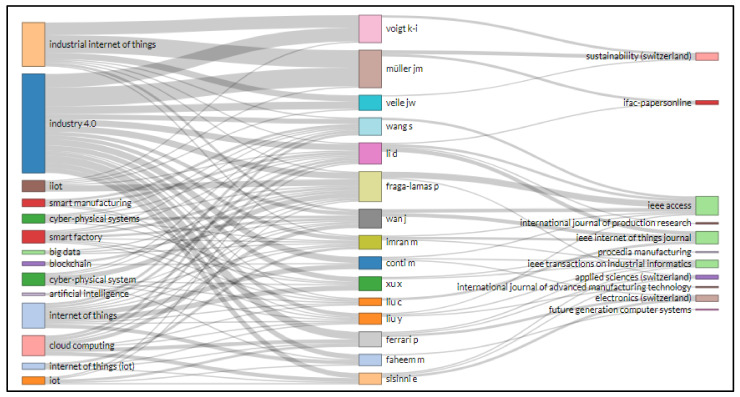
Three field plot author keyword (**left**), author (**middle**), and source (**right**).

Figure 19 shows the illustration for research related to IoT in Ind 4.0 literature. The left side of the diagram presents the author keyword; on the right side, journal names are shown, and in the middle, the author’s names. The scrutiny of a diagram can show which author keyword of IoT in Ind 4.0 had been used most recurrently by diverse authors and journals. For example, four keywords, i.e., “industrial internet of things”, “industry 4.0”, “internet of things”, and “cloud computing”, were utilized by Voigt K. I., Muller J. M., Fraga-Lamas P. and Wan J. and published in Sustainability, IEEE Access, and IEEE Internet of Things Journal.

**Figure 20:**
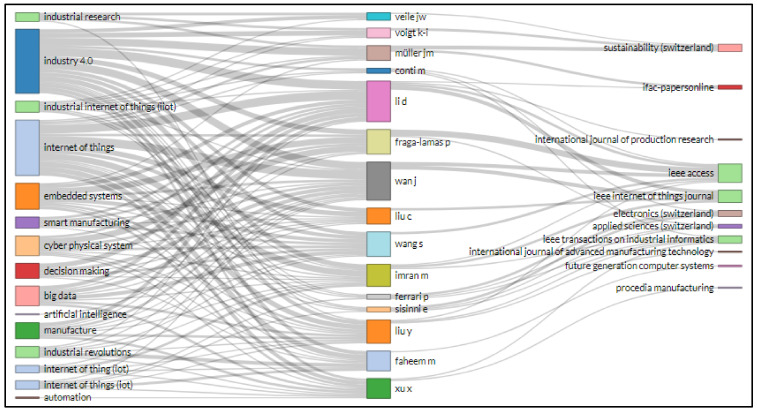
Three field plot keywords plus (**left**), author (**middle**), and source (**right**).

Similarly, Figure 20 shows the illustration for research related to IoT in Ind 4.0 literature. Again, the left side of the diagram presents the index keyword; on the right side, journal names are shown, and in the middle, authors’ names. In the visualization of the uppermost index keyword, authors and sources specified that there were index keywords, including “industry 4.0”, “internet of things”, embedded systems”, and “big data”, that had been utilized mostly by Li D., Wan J., Wang S., Liu Y. and Xu X. and frequently published in Sustainability, IEEE Access, and IEEE Internet of Things Journal.

**Figure 21:**
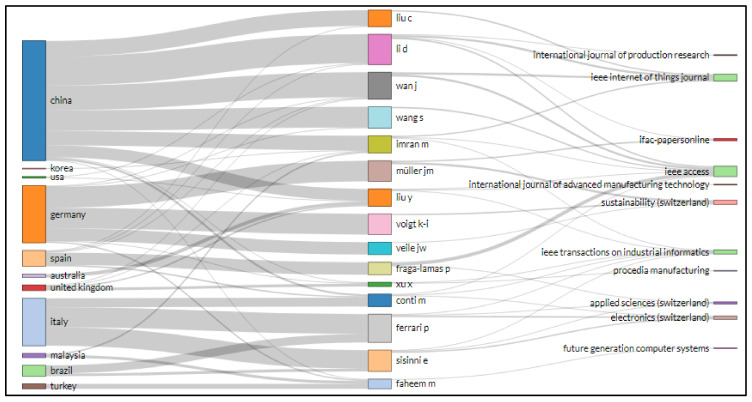
Three-field plot of country (**left**), author (**middle**), and source (**right**).

Figure 21 shows the illustration for research related to IoT in Ind 4.0 performed by different countries and published in different journals. The larger size of the rectangle signifies that China performed well with significant contributions of Li D., Liu C., Wan J., Wang S. and Imran M., and these authors from China mainly published their research related to IoT in Ind 4.0 in IEEE Access. On the other hand, the rectangle size indicates that Germany also performed well; Muller J. M., Liu Y., Voigt K. I. and Veile J. W. published their research related to IoT in Ind 4.0, mainly in Sustainability and IEEE Access. Italy has main authors such as Ferrao P., Conti M. and Sisinni E, who published mainly in Electronics (Switzerland).

## 5. Latest Trends, Summary, Future Roadmaps, and Concluding Remarks

### 5.1. Latest Trends in IoT in Ind 4.0

**Figure 22:**
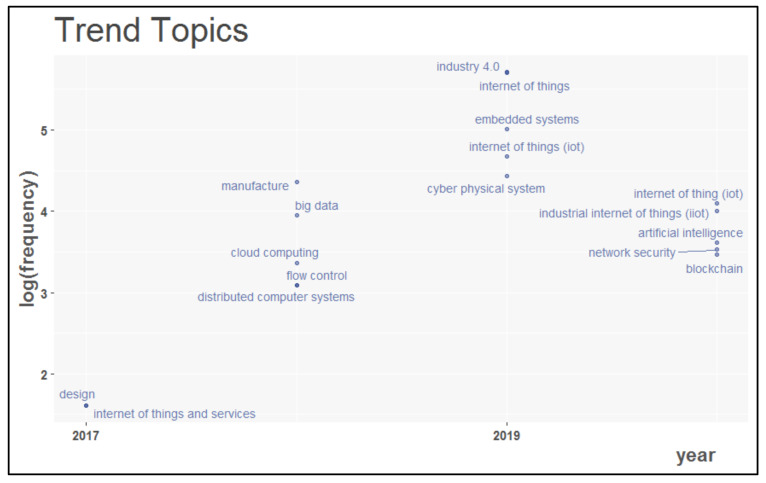
Latest trends based on index keywords in IoT in Ind 4.0 research.

**Figure 23:**
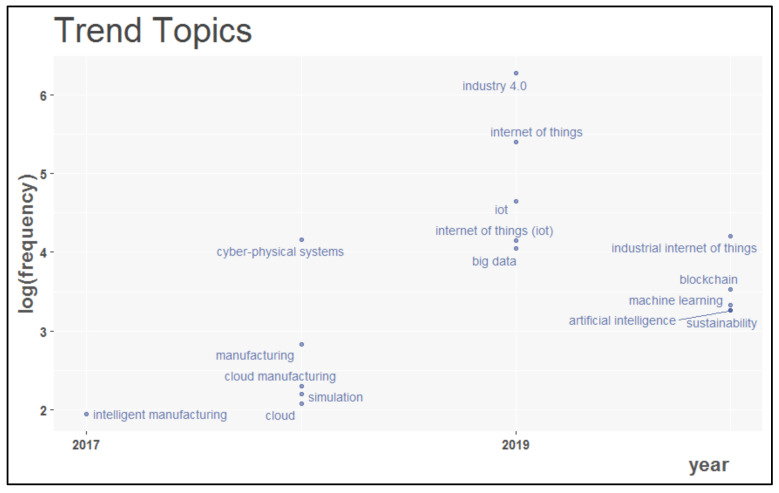
The latest trends are based on author keywords.

In addition, the evolutionary trend of IoT in different types of industrial applications was analyzed using index keywords and author’s keywords, shown in Figure 22 and Figure 23, respectively. For this analysis, the period considered was from 2014 to 2020. While studying the base of index keywords, it was observed that in the year 2017, IoT was integrated into industrial design applications.

In 2018, this revolutionary technology was mingled with many other evolving technologies, such as big wise data, cloud computing, distributed systems, and manufacturing. With further development, it was placed in cyber–physical systems and embedded systems to benefit various applications in Industry 4.0. Recent growth in the industrial usage of the Internet of Things has introduced the Industrial Internet of Things (IIoT), amalgamated into various industrial domains. IoT in conjunction with AI and Blockchain provides more secure industrial applications. Using author keywords, IoT is closely integrated with many other advanced technologies such as machine learning, intelligent manufacturing, cloud computing, and simulation to provide sustainable solutions in the Industry 4.0 ecosystem.

### 5.2. Summary and Future Roadmaps for IoT in Ind 4.0

The unavailability of systematic evolutionary development of IoT in the Industry 4.0 environment for the considered period is the biggest motivation behind this novel piece of work. On one side, this work has provided insight into the field and has offered possible future research directions. To avoid any misinterpretation or confusion, universally recognized keywords related to the domain were used to gather the data. The present systematic review portrays the role played by IoT in Industry 4.0 ecosystem in coordination with other advanced technologies. It has been observed that IoT plays a key role in many industrial processes in coordination with other advanced technologies such as artificial intelligence, machine learning, big data, cloud computing, cyber–physical systems, and intelligent manufacturing. This firm collaboration has benefitted many industrial operations and processes. This systematic review has derived the following possible research directions for scientists and engineers:

- In-depth understanding of IoT and Industry 4.0 ecosystem and applications.
- Optimal interfacing of IoT with other advanced technologies.
- Standardization of protocols for smart industrial processes.
- Developing smart embedded systems.
- Integration of IoT to cater to sustainable development of industrial applications.
- Implementing optimal, productive, flexible, and customized industrial solutions.

IoT is further expected to play a more vital role in monitoring, predictive maintenance, inventory management, quality control, supply chain optimization, and plant safety.

### 5.3. Limitations of Analysis for IoT in Ind 4.0

While undertaking a theoretical assessment, the current study has obvious limitations. The study’s data gathering was constrained because it relied solely on Scopus for its literature review, and conference proceedings were not considered for analysis. As a result, the paper’s reach can be expanded by including articles from other databases, such as Google Scholar. Comprehensive bibliometric research was presented to distinguish between ISI-WoS and Scopus results. According to the bibliometric study, around 2/3 of the referred documents were found in both databases, whereas 1/3 were only found in one. The citation effect of the core papers in both databases was larger, but the influence of the peripheral publications in just one database must not be overlooked because some high-impact articles may be identified among them. Many Scopus publications were not detected in WoS. However, they were documented in ISI Transactions since Scopus includes an aggregate reference to the conference proceedings without a descriptor of each contribution and, hence, of the names or addresses of their authors [88,89].

### 5.4. Concluding Remarks

In this work, we made an effort to contribute to Industry 4.0 by studying the evolution of IoT in the various processes, applications, subsystems, and other areas. This much-needed work was carried out in this most significant domain using bibliometric and content analyses. We gathered eight hundred and ninety-one articles from 2014 to 2020 from the most authentic Scopus database to carry out this research work. In this study, we aimed to conduct a bibliometric investigation and visual valuation of IoT in Ind 4.0 evolution to link the disparity in the available literature analysis and research directions.

The paper “Intelligent manufacturing in the context of Industry 4.0: a review” by Zhong et al., (2017) [59] was rated first with 754 citations, followed by “Industry 4.0: state of the art and future trends” by Xu et al., (2018) [32]. The integration of IoT in multiple processes revolutionized multiple facets in the Ind 4.0 environment, dedicated to different aspects such as design, production processes, manufacturing, economy, services, sustainability, business strategies, supply chain, data security and communication, and so on in various types of organizations, such as SMEs and the electrical and machine industries. Integrating IoT with other sophisticated technologies such as AI, big data, cloud computing, fog computing, blockchain, and CPS has resulted in a significant advancement. CPS has been identified as a crucial participant in the IoT era of industrial development.

With 1629 citations, the “International Journal of Production Research” was ranked #1, and the first paper on IoT in Industry 4.0 was published in 2018. The IEEE Access ranked first with 53 articles and second with 1470 total citations. Wan J. ranked first, based on a score of 1031, second based on a score of 132, and third with eight NoA. Wan J. is a researcher at Guangzhou’s South China University of Technology’s School of Mechanical and Automotive Engineering. In addition, 2512 authors contributed to one article with a proportion of authors of 0.8992, while 227 authors produced two papers with a proportion of 0.081. Three reports were written by 46 authors, resulting in a 0.016 ratio of authors. The South China University of Technology in Guangzhou, China, was placed first with a score of 788, 4, and 17. Beihang University in Beijing, China, came in second place with a score of 439 and first place with a score of 220. With 4820 total citations, 254 articles, and 178 total link strengths, the United States was identified as the most prolific and influential country, ranking fourth in average article citations (19). Singapore was in the first place, with an average of 82 article citations.

The author keyword dynamics revealed that ‘Industry 4.0’ appeared first in 2014 with the application of IoT in Ind 4.0 with an overall appearance of 528, followed by the ‘internet of things’ in 2015, and three times with a total count of 220 up to 2020. The terms have much scope for research with IoT in industry 4.0, such as ‘industrial internet of things’ with an appearance of only 17 times, ‘cyber–physical systems’ (16 times), and ‘big data’ (14 times). ‘Smart manufacturing’, ‘smart factory’, and ‘cloud computing’ with appearances of 13, 11, and 10 also have much potential, and these are termed as research gaps in the application of IoT in Ind 4.0. Similar results were revealed by index keyword dynamics, and a central gap exists in the terms ‘decision making’ and ‘industrial revolution’ while using IoT in Ind 4.0.

In text data from IoT in Industry 4.0, i.e., data taken from abstracts and titles of supporting documents, the word “application” appears 282 times in the top 25 phrases, having a relevance score of 0.407. On the other hand, the term “design methodology approach” was at the top of the list, with an RS of 8.432 and an occurrence of 34. Sankey diagrams are commonly utilized as representations of energy or material flows in a variety of networks and methodologies. They are numerical representations of flows, linkages, and transitions. Sankey diagrams create guided and weighted designs that keep the flow going. Voigt K. I., Muller J. M., Fraga-Lamas P. and Wan J. used four keywords: “industrial internet of things”, “industry 4.0”, “internet of things”, and “cloud computing” in articles published in Sustainability, IEEE Access, and IEEE Internet of Things Journal.

It has been observed that over time, IoT started to amalgamate with other advanced technologies such as Artificial Intelligence, Cloud Computing, Bigdata, Distributed Systems, Embedded Systems, Cyber-physical Systems, Blockchain, and IIoT. This integration benefitted several Ind 4.0 application domains, such as design, manufacturing, robotics, supply-chain, etc. For example, manufacturing evolved as intelligent manufacturing. As most of the data are transmitted in cyberspace, more secure and reliable IoT integrated secure applications are deployed in the Ind 4.0 ecosystem. A huge volume of data gathered from various industrial applications is stored in the cloud, which reduces memory requirements and initiates optimal usage of the resources. The inclusion of IoT in various industrial processes caused energy efficiency, economical products and services, better quality, efficient decisions, and reduced equipment downtime.

The research hotspots and gaps related to IoT in Ind 4.0 show a prominent scope for future young researchers. However, such as in every study, this investigation also suffered from some inherent limitations. For example, many papers in other languages were not considered in the present research. There are also many articles not available in this database that were not part of this study. Nevertheless, the significant results of this bibliometric analysis can be used for future research with some novel applications in various research areas. Furthermore, the study will allow researchers in IoT in Ind 4.0 to expand quickly in comprehending the development status and pattern.

## Figures and Tables

**Table 1:** IoT in Ind 4.0 data assortment steps.

Exploration Steps	Question on Scopus	Description	Count
1	TITLE-ABS-KEY	(“Internet of Things”) OR (“IoT”) OR (“Internet of Things (IoT)”) OR (“Industrial Internet of Things (IIoT)”) OR (“IIoT”) AND (“Industry 4.0”)	3907
2	AND (LIMIT-TO)	(LANGUAGE, “English”)	3810
3	AND (EXCLUDE)	(PUBYEAR,2021)	3433
4	AND (LIMIT-TO)	(DOCTYPE, “ar”)	1014
5	AND (EXCLUDE (SUBJAREA))	(“PSYC”) OR (“EART”) OR (“HEAL”) OR (“PHAR”) OR (“IMMU”) OR (“MEDI”)	891

**Table 2:** Inclusive and exclusive prerequisite conditions.

Parameter of Selection of a Paper	Inclusion Criterion	Exclusion Criterion	Rationale for Inclusion–Exclusion
Language	English	Rest all languages	The researchers and the preponderance of readership can readily understand English.
Year	2014 to 2020	Publications before 2014 and after 2020	During this time, the majority of studies were carried out. There were only a few before 2014, and investigations after 2020 are in process.
Open Access	All	No Exclusion	All papers were essential.
Author Name	All		Discrimination on these grounds made no sense.
Subject Area	All	Yes Exclusion	Not every subject area was important for evaluating IoT articles in Ind 4.0.
Keyword	All	No Exclusion	The keywords connected directly to the study were imperative.
Publication Status	All		All papers had to be added.
Source Title	All		All journal titles were moderately pertinent.
Affiliation	All		This structure did not violate the assortment criterion.
Funding Sponsor	All		This structure did not violate the assortment criterion.
Country	All		Publication from each country had its implications.
Source Type	All	Yes Exclusion	Limited to journals, others were not entirely relevant.

**Table 3:** Top ten articles of IoT in Ind 4.0 based on total citations.

Article Authors	ŤĈ	Rank on ŤĈ	ŤŁŜ	Rank on ŤŁŜ	ƤŜŷ	Ĉŷ	ŤĈ/ŷ	Rank on ŤĈ/ŷ	Ref.
Zhong R.Y., Xu X., Klotz E., Newman S.T.	754	1	9	4	2017	3	251.3	3	[59]
Xu L.D., Xu E.L., Li L.	686	2	9	4	2018	2	343	1	[32]
Wollschlaeger M., Sauter T., Jasperneite J.	674	3	11	3	2017	3	224.7	4	[61]
Kang H.S., Lee J.Y., Choi S., Kim H., Park J.H., Son J.Y., Kim B.H., Noh S.D.	524	4	9	4	2016	4	131	7	[62]
Hofmann E., Rüsch M.	514	5	15	1	2017	3	171.3	5	[60]
Wan J., Tang S., Shu Z., Li D., Wang S., Imran M., Vasilakos A.V.	367	6	15	1	2016	4	91.75	10	[63]
Frank A.G., Dalenogare L.S., Ayala N.F.	320	7	7	7	2019	1	320	2	[20]
Chen B., Wan J., Shu L., Li P., Mukherjee M., Yin B.	314	8	2	10	2017	3	104.7	8	[64]
Tao F., Zhang M.	310	9	3	9	2017	3	103.3	9	[65]
Moeuf A., Pellerin R., Lamouri S., Tamayo-Giraldo S., Barbaray R.	276	10	4	8	2018	2	138	6	[66]

ŤĈ: Total citations; ŤŁŜ: total link strength; ƤŜŷ: Publication start year; Ĉŷ: Citable years; ŤĈ/ŷ: Total citations yearly.

**Table 4:** Contributions of top-cited 25 articles: Technologies, integration, and domains.

Year	Technologies for Possible Integration	Domains	Evolution of Technology(s)	Ref.
2017	IoT, AI, Cloud Computing and CPS	Intelligent Manufacturing	No	[59]
2018	IoT, Cloud Computing and CPS	Security and Privacy	No	[32]
2017	IoT, CPS and ICT	Process Automation	No	[61]
2016	IoT	Smart Manufacturing	Yes	[62]
2017	IoT, CPS, and IoS	Smart Factory	No	[60]
2019	IoT, CPS, IIoT	Customizable Services	Yes-Partially	[67]
2019	IoT, Cloud Computing, and Big Data	Smart Manufacturing and Smart Supply Chain	No	[20]
2017	IoT, Cloud Computing, and Big Data	Manufacturing	No	[64]
2017	IoT, AI, Big Data	Digital Twin Shop-floor	NO	[65]
2017	IoT, CPS, M2M Communication	Production Planning in SMEs	Yes-Partially	[66]
2016	IoT	Lean Manufacturing	Yes	[68]
2018	IoT, CPS, Cloud Computing, and Simulation	Economic Stability and Sustainability	No	[69]
2018	IIoT, CPS	Industrial Connectivity	No	[70]
2018	IoT	Sustainability	No	[71]
2018	IoT	Circular Economy	No	[72]
2017	IoT and CPS	Business Strategies	No	[73]
2018	ICT, IoT, and CPS	Lean Manufacturing	No	[74]
2018	IoT, Big Data, and CPS	Resource Optimization	No	[75]
2017	IIoT	Electrical and Machine Industry	No	[76]
2017	IIoT	Supply Chain	Yes-Partially	[77]
2019	IoT, CPS, Big Data	Sustainability	No	[78]
2018	IIoT and Fog Computing, blockchain	Security and Communication Delays	No	[79]
2017	IoT	Smart Factory	No	[80]
2016	CPS	Manufacturing	No	[81]
2015	IoT	SMEs	No	[82]

**Table 5:** Top Ten Journals based on selected criteria.

Journals	ŤĈ	Rank on ŤĈ	NoA	Rank on NoA	ŤŁŜ	Rank on ŤŁŜ	ÄĈ	Rank on ÄĈ	ƤŜŷ	ĬF	CS
International Journal of Production Research	1629	1	17	6	89	1	96	4	2018	4.577	7.6
IEEE Access	1470	2	53	1	52	5	28	9	2016	3.745	9.0
Computers in Industry	1247	3	17	6	71	2	73	5	2016	3.954	10
Procedia Manufacturing	1075	4	20	5	61	4	54	6	2015	-	1.9
Sustainability (Switzerland)	973	5	28	2	64	3	35	7	2017	2.576	3.9
Engineering	939	6	3	8	36	6	313	1	2017	6.495	8.2
IEEE Transactions on Industrial Informatics	859	7	28	2	30	8	31	8	2017	9.112	13.9
IEEE Industrial Electronics Magazine	710	8	3	8	27	9	237	3	2017	13.593	16.4
International Journal of Precision Engineering and Manufacturing Green Technology	602	9	2	10	15	10	301	2	2016	4.171	7.5
IFAC-PapersOnLine	601	10	28	2	33	7	21	10	2016	-	1.6

ŤĈ: Total citations; NoA: Number of articles; ŤŁŜ: total link strength; ÄĈ: Average citations per document; ƤŜŷ: Publication start year; ĬF: Impact factor (2019); CS: CiteScore.

**Table 6:** Top Ten Authors based on ŤĈ, NoA, ŤŁŜ, and ÄĈ.

Author	ŤĈ	Rank on ŤĈ	NoA	Rank on NoA	ŤŁŜ	Rank on ŤŁŜ	ÄĈ	Rank on ÄĈ	h_index	g_index	m_index	ƤŜŷ
Wan J.	1031	1	8	3	132	2	129	8	7.000	8.000	1.167	2016
Li D.	946	2	11	2	142	1	86	9	9.000	11.000	1.500	2016
Xu L.D.	907	3	4	5	43	6	227	6	4.000	4.000	1.000	2018
Xu X.	903	4	5	4	50	4	181	7	4.000	5.000	0.571	2015
Zhong R.Y.	803	5	3	6	50	4	268	5	3.000	3.000	0.600	2017
Müller J.M.	732	6	15	1	84	3	49	10	1.000	1.000	0.200	2017
Li L.	689	7	2	7	33	10	345	1	1.000	1.000	0.200	2017
Jasperneite J.	680	8	2	7	35	7	340	2	9.000	15.000	1.800	2017
Sauter T.	680	8	2	7	35	7	340	2	2.000	2.000	0.500	2018
Wollschlaeger M.	680	8	2	7	35	7	340	2	1.000	1.000	0.250	2018

ŤĈ: Total citations; NoA: Number of articles; ŤŁŜ: total link strength; ÄĈ: Average citations per document; ƤŜŷ: Publication start year.

**Table 7:** Top ten organizations and nations.

Organizations	ŤĈ	Rank on ŤĈ	NoA	Rank on NoA	ŤŁŜ	Rank on ŤŁŜ	ÄĈ	Rank on ÄĈ
South China University of Technology, Guangzhou, China	788	1	4	1	17	1	197	2
Beihang University, Beijing, China	439	2	2	3	1	8	220	1
Universidade Federal Do Rio Grande Do Sul, Brazil	359	3	3	2	2	4	120	4
California State University, United States	267	4	2	3	4	2	134	3
Campus Universitário De Santiago, Portugal	233	5	2	3	1	8	117	5
North-West University, South Africa	224	6	2	3	2	4	112	6
Friedrich-Alexander University Erlangen-Nürnberg, Germany	213	7	2	3	3	3	107	7
Ierg, University College Cork, Ireland	189	8	2	3	2	4	95	8
Federal University of Rio Grande Do Sul, Porto Alegre, Brazil	164	9	2	3	1	8	82	9
Vit University, Vellore, India	147	10	2	3	2	4	74	10
**Nations**	**ŤĈ**	**Rank** on **ŤĈ**	**NoA**	**Rank** **on NoA**	**ŤŁŜ**	**Rank** **on ŤŁŜ**	**ÄĈ**	**Rank** **on ÄĈ**
Germany	4172	1	90	2	337	1	46	2
United Kingdom	3247	2	66	6	287	4	49	1
United States	3039	3	95	1	313	2	32	6
China	2951	4	83	4	250	5	36	5
Italy	1483	5	84	3	179	7	18	8
India	1440	6	82	5	299	3	18	9
Brazil	1205	7	44	8	214	6	27	7
Austria	1094	8	25	10	115	10	44	3
Spain	1093	9	65	7	131	9	17	10
France	1062	10	28	9	132	8	38	4

ŤĈ: Total citations; NoA: Number of articles; ŤŁŜ: total link strength; ÄĈ: Average citations per document.

**Table 8:** Author keyword dynamics from 2014 to 2020.

Year	2014	2015	2016	2017	2018	2019	2020	Sum	Avg.
Industry 4.0	1	8	21	44	93	142	219	528	132
Internet of Things	0	3	10	23	52	50	82	220	55
IoT	0	2	4	4	17	35	42	104	26
Industrial Internet of Things	0	0	1	3	9	20	34	67	17
Cyber-Physical Systems	0	1	6	9	17	16	15	64	16
Internet of Things (IoT)	0	0	4	1	11	16	31	63	16
Big Data	0	1	1	4	10	20	21	57	14
Smart Manufacturing	0	1	2	8	13	13	16	53	13
Smart Factory	0	0	4	7	10	10	14	45	11
Cloud Computing	0	0	2	3	9	11	16	41	10

**Table 9:** Index keyword dynamics from 2014 to 2020.

Year	2015	2016	2017	2018	2019	2020	Sum	Avg.
Industry 4.0	0	8	9	72	86	127	302	50
Internet of Things	3	18	17	86	77	99	300	50
Embedded Systems	3	12	22	37	38	38	150	25
Internet of Things (IoT)	1	6	6	25	29	40	107	18
Cyber Physical System	0	7	14	20	29	14	84	14
Manufacture	1	7	17	18	16	19	78	13
Industrial Revolutions	1	6	4	17	13	34	75	13
Decision Making	1	7	5	11	9	30	63	11
Internet of Thing (IoT)	0	2	3	11	12	32	60	10
Industrial Internet of Things (IioT)	0	0	0	1	4	50	55	9

**Table 10:** Top 25 text data terms.

Rank	Term	Occ	RS	Rank	Term	Occ	RS
1	Application	282	0.407	1	Design Methodology Approach	34	8.432
2	Study	215	0.856	2	Originality Value	36	7.665
3	Network	210	1.230	3	IoT Device	41	3.906
4	Solution	197	0.485	4	Manager	46	2.262
5	Research	195	0.686	5	Protocol	60	2.225
6	Device	183	1.523	6	Gap	46	1.896
7	Manufacturing	179	0.183	7	Sustainability	46	1.863
8	Service	165	0.371	8	Factor	72	1.649
9	Cyber-Physical System	164	0.436	9	New Technology	42	1.606
10	Time	155	0.291	10	Researcher	34	1.590
11	Industrial Internet	143	0.877	11	Innovation	60	1.565
12	Integration	141	0.246	12	Organization	68	1.565
13	Requirement	141	0.392	13	Reliability	50	1.548
14	Company	137	1.377	14	Device	183	1.523
15	Architecture	133	0.547	15	Deployment	52	1.511
16	Communication	122	1.372	16	Algorithm	69	1.435
17	Control	120	0.540	17	Literature	84	1.410
18	Production	119	0.252	18	Smart City	34	1.409
19	Machine	118	0.365	19	Scenario	89	1.407
20	Problem	115	0.280	20	Company	137	1.377
21	Sensor	114	0.882	21	Communication	122	1.372
22	Platform	110	0.562	22	Number	84	1.346
23	Big Data	108	0.423	23	User	62	1.304
24	Change	104	0.650	24	Monitoring	88	1.302
25	Fourth Industrial Revolution	99	0.248	25	Business	45	1.287

**Table 11:** Title words dynamics of IoT in Ind 4.0.

Year	Industry	Smart	Manufacturing	Industrial	Internet	Things	Systems	IoT	Data	System
2014	2	0	0	0	1	1	0	0	0	0
2015	8	3	5	4	3	4	3	2	2	1
2016	22	9	13	9	11	6	5	5	3	6
2017	34	16	13	6	6	6	14	7	4	7
2018	77	31	30	24	23	21	18	19	15	11
2019	96	39	41	26	26	26	27	18	23	14
2020	155	52	47	58	42	38	26	39	23	31
Sum	394	150	149	127	112	102	93	90	70	70
Avg.	99	38	37	32	28	26	23	23	18	18

**Table 12:** Abstract words dynamics of IoT in Ind 4.0.

Year	Industry	Data	Manufacturing	Industrial	Internet	IoT	Systems	Technologies	Smart	Things
2014	0	0	0	0	2	0	2	0	0	1
2015	20	16	23	10	15	3	12	12	19	11
2016	76	24	61	48	39	28	44	31	36	28
2017	137	48	139	54	64	35	81	51	66	56
2018	378	180	210	176	163	139	161	151	155	140
2019	435	311	226	219	223	223	198	203	172	187
2020	729	506	332	387	318	393	286	291	285	285
Sum	1775	1085	991	894	824	821	784	739	733	708
Avg.	444	271	248	224	206	205	196	185	183	177

## Data Availability

Not applicable.
